# Supplementary material for: Phase separation on cell surface facilitates bFGF signal transduction with heparan sulphate
Source: Nat Commun. 2022 Mar 2;13:1112. doi: 10.1038/s41467-022-28765-z (PMC8891335; doi:10.1038/s41467-022-28765-z)
Supplement: Supplementary file 1 — Supplementary Information [file 41467_2022_28765_MOESM1_ESM.pdf]

Supplementary Materials

Phase separation on cell surface facilitates bFGF signal transduction with heparan sulphate

Song Xue[1,2,4], Fan Zhou[1,4], Tian Zhao[1,4], Huimin Zhao[1], Xuewei Wang[1], Long Chen[1], Jin-ping Li[2,3], Shi-Zhong Luo[1*]

[1]Beijing Key Laboratory of Bioprocess, College of Life Science and Technology, Beijing University of Chemical Technology, Beijing, 100029, China

[2]Beijing Advanced Innovation Centre for Soft Matter Science and Engineering, Beijing University of Chemical Technology, Beijing, 100029, China

[3] Department of Medical Biochemistry and Microbiology, Uppsala University, 75123, Uppsala, Sweden

[4] These authors contributed equally: Song Xue, Fan Zhou and Tian Zhao

[*]e-mail: luosz@mail.buct.edu.cn

Figures

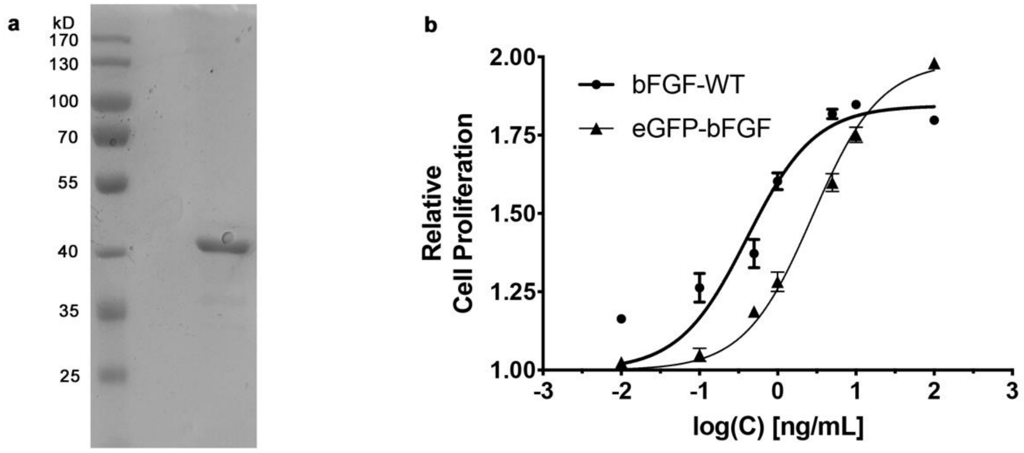

Supplementary Fig. 1 Characterization of eGFP-bFGF. **a**, SDS-PAGE showed the molecular weight and purity of eGFP-bFGF. **b,** Expressed eGFP-bFGF has similar activity as commercially tag-free bFGF, n=3 biologically independent samples, data are presented as mean values +/- SEM.

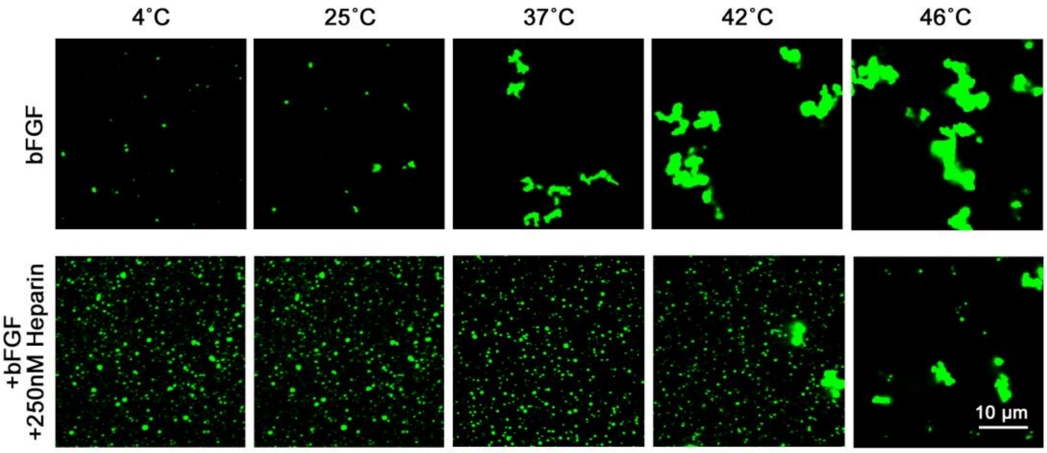

Supplementary Fig. 2 Phase separation of bFGF with heparin increases the thermo stability of bFGF. Aggregates formed in the solution of 5 μM of bFGF as the temperature increased from 4 to 46 ˚C. With 250 nM of heparin added, bFGF existed in a phase separation status at the temperature from 4 to 42 ˚C and aggregates formed at 46 ˚C. Scale bar=10 μm.

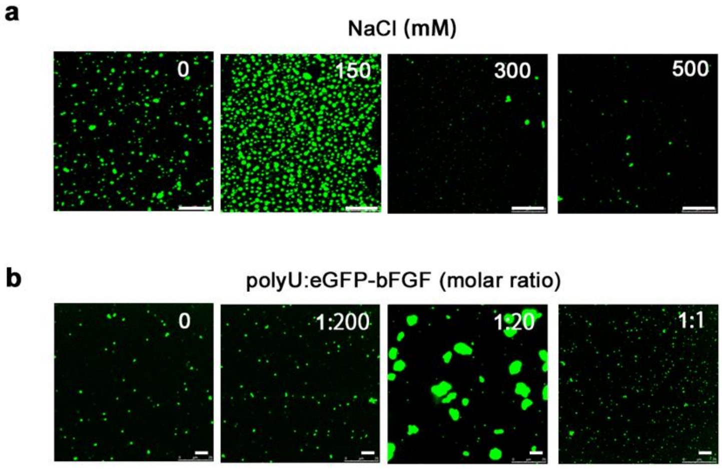

Supplementary Fig. 3 Phase separation of eGFP-bFGF was driven by electrostatic interactions. **a**, LLPS of eGFP-bFGF with heparin under various concentrations of NaCl. Scale bar=5 μm. **b**, polyU induced LLPS od eGFP-bFGF with a dose-dependent manner. Scale bar=10 μm.

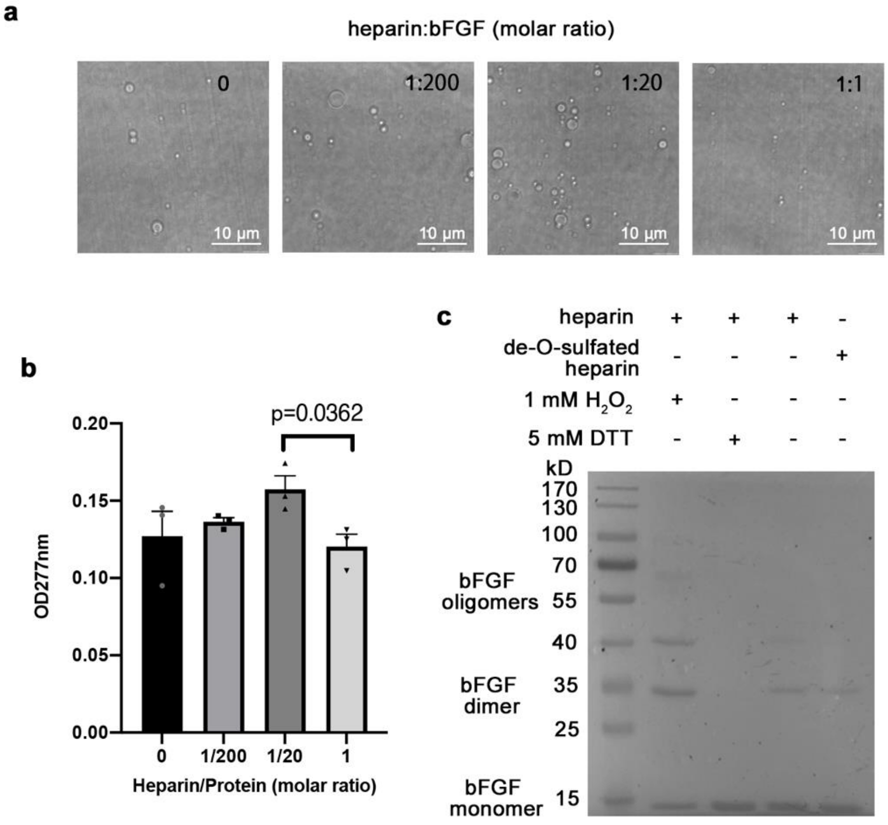

Supplementary Fig. 4 Phase separation and assemble status of tag-free bFGF. **a**, phase separation of bFGF was firstly promoted and then inhibited as heparin concentration increased. Scale bar=10 µm. **b**, the corresponding turbidity the samples in **a**, n=3 biologically independent samples data are presented as mean values +/- SEM. Comparisons among groups were performed using two-tailed unpaired t-test. **c**, Non-reduced SDS-PAGE of the tag-free bFGF with different oxidative conditions and heparin concentrations, showing its assembling status.

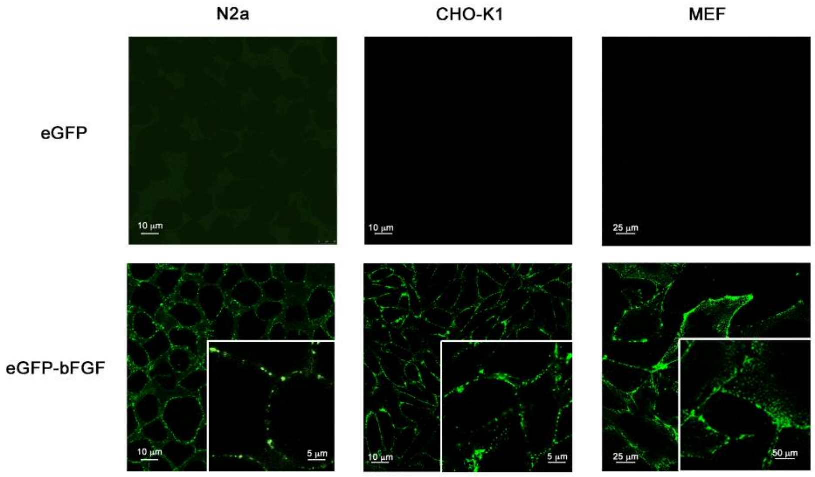

Supplementary Fig. 5 Confocal microscopy images of bFGF phase separation on cell surface, with eGFP as control. Scale bar=10 µm for N2a and CHO-K1 cells and 25 µm for MEF cells.

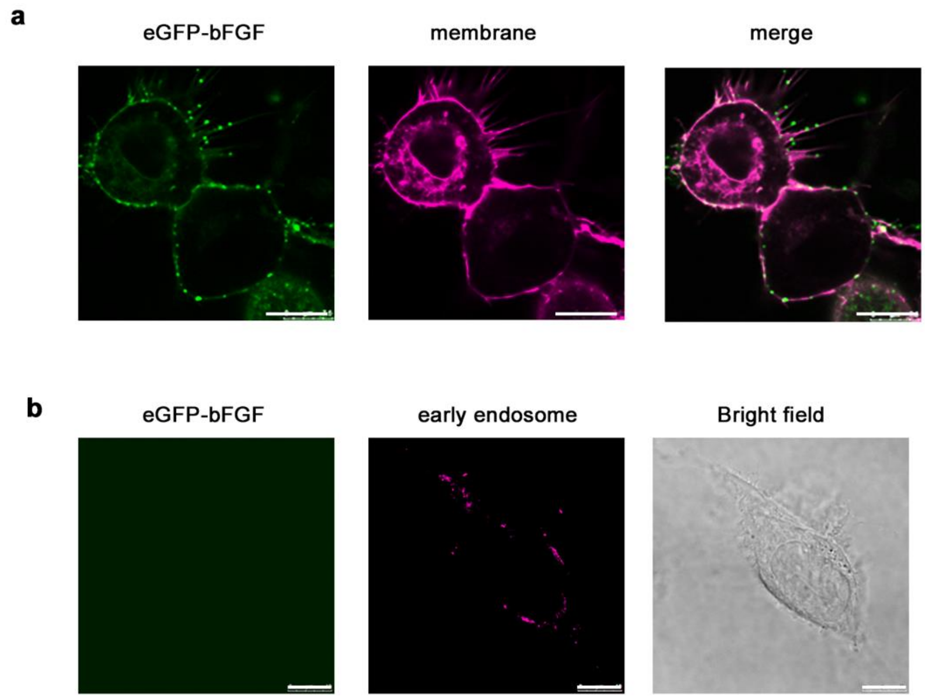

Supplementary Fig. 6 The condensates of eGFP-bFGF are on the outer membrane. **a**, confocal microscopy of eGFP-bFGF condensates (green) and cell membrane (magenta). Scale bar=10 μm. **b**, early endosome staining within 5 min of condensates formation, the condensates were washed away and not observed in early endosome. Scale bar=10 μm.

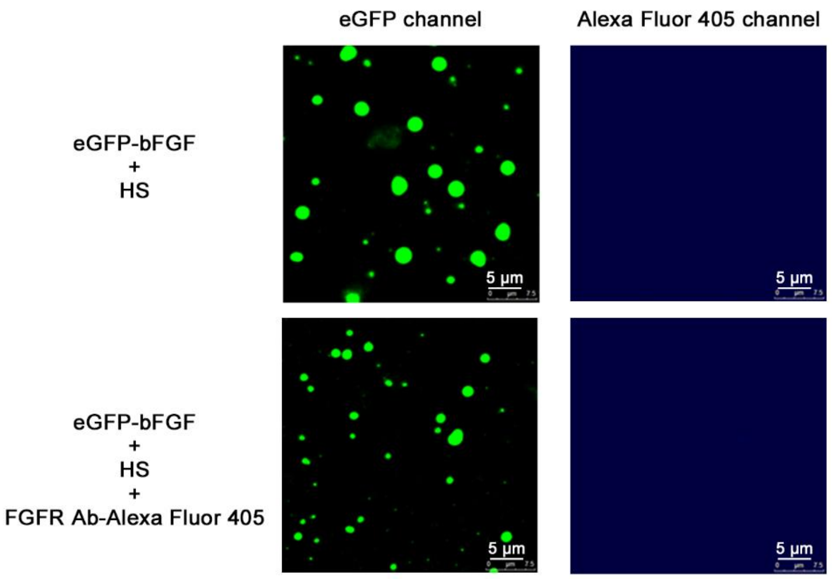

Supplementary Fig. 7 The FGFR antibody has no effect on bFGF condensation or distribution. The antibody did not co-condensate with bFGF. Scale bar=5 μm.

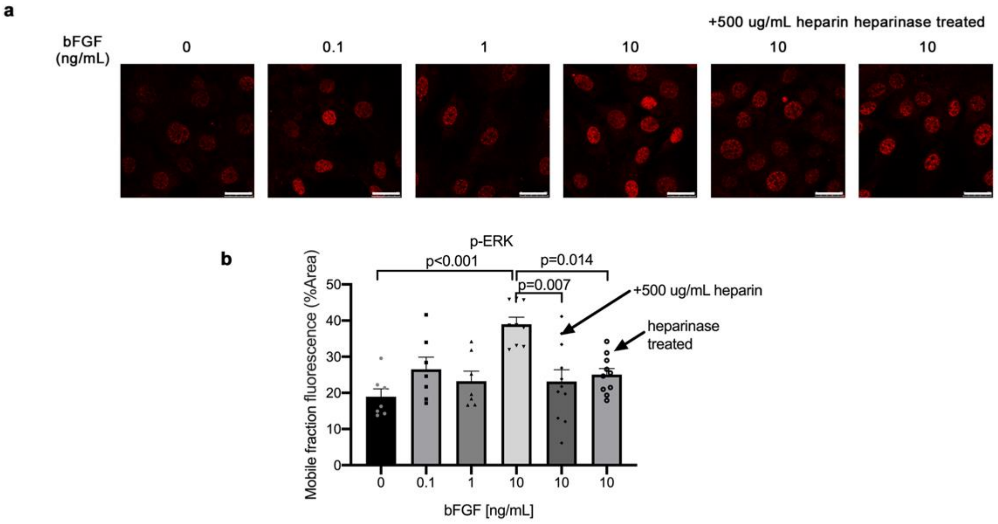

Supplementary Fig. 8 Staining of p-ERK and statistics of the intensity. **a**, images of p-ERK with different concentration of bFGF and heparin or heparinase treated cells. Scale bar=25 μm. **b**, the average intensity of p-ERK in each cell from the samples, n=7,7,7,9,11 and 10 cells depending on the numbers of cells in the view, data are presented as mean values +/- SEM. Comparisons among groups were performed using ordinary two-way ANOVA test.

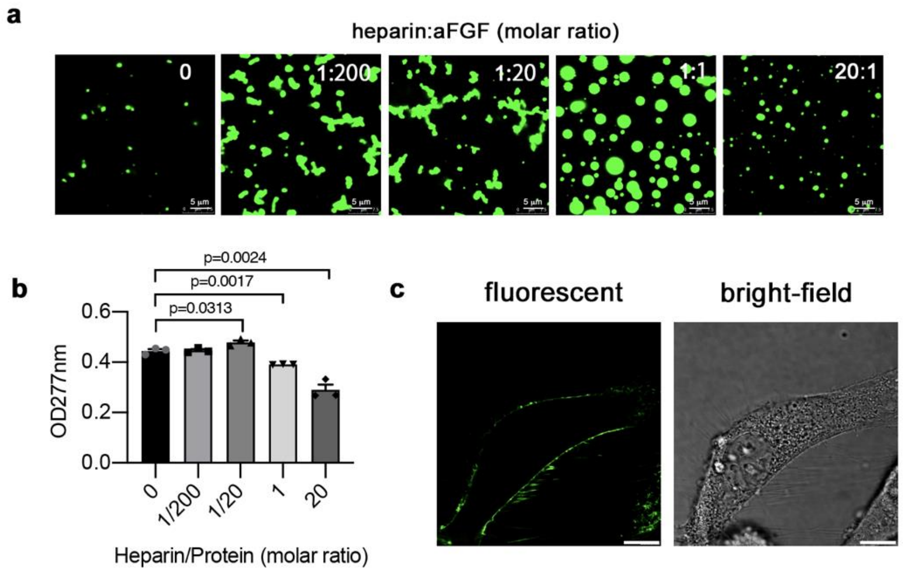

Supplementary Fig. 9 Phase separation of acidic fibroblast growth factor (aFGF). **a**, phase separation of aFGF was firstly promoted and then inhibited as heparin concentration increased. Scale bar=5 µm. **b**, the corresponding turbidity the samples in **a**. n=3 biologically independent samples, data are presented as mean values +/- SEM. Comparisons among groups were performed using two-tailed unpaired t-test. **c**, phase separation of aFGF on MEF cell surface. Scale bar=10 µm.
