# Supplementary material for: Phase separation on cell surface facilitates bFGF signal transduction with heparan sulphate
Source: Nat Commun. 2022 Mar 2;13:1112. doi: 10.1038/s41467-022-28765-z (PMC8891335; doi:10.1038/s41467-022-28765-z)
Supplement: Supplementary file 2 — Reporting Summary [file 41467_2022_28765_MOESM2_ESM.pdf]

nature portfolio

Corresponding author(s): Shi-Zhong Luo

Last updated by author(s): Jan 15, 2022

# Reporting Summary

Nature Portfolio wishes to improve the reproducibility of the work that we publish. This form provides structure for consistency and transparency in reporting. For further information on Nature Portfolio policies, see our Editorial Policies and the Editorial Policy Checklist.

## Statistics

For all statistical analyses, confirm that the following items are present in the figure legend, table legend, main text, or Methods section.

| n/a | Confirmed | |
|---|---|---|
| ☐ | ☒ | The exact sample size ($n$) for each experimental group/condition, given as a discrete number and unit of measurement |
| ☐ | ☒ | A statement on whether measurements were taken from distinct samples or whether the same sample was measured repeatedly |
| ☐ | ☒ | The statistical test(s) used AND whether they are one- or two-sided<br>*Only common tests should be described solely by name; describe more complex techniques in the Methods section.* |
| ☐ | ☒ | A description of all covariates tested |
| ☐ | ☒ | A description of any assumptions or corrections, such as tests of normality and adjustment for multiple comparisons |
| ☐ | ☒ | A full description of the statistical parameters including central tendency (e.g. means) or other basic estimates (e.g. regression coefficient) AND variation (e.g. standard deviation) or associated estimates of uncertainty (e.g. confidence intervals) |
| ☐ | ☒ | For null hypothesis testing, the test statistic (e.g. $F$, $t$, $r$) with confidence intervals, effect sizes, degrees of freedom and $P$ value noted<br>*Give P values as exact values whenever suitable.* |
| ☒ | ☐ | For Bayesian analysis, information on the choice of priors and Markov chain Monte Carlo settings |
| ☒ | ☐ | For hierarchical and complex designs, identification of the appropriate level for tests and full reporting of outcomes |
| ☒ | ☐ | Estimates of effect sizes (e.g. Cohen's $d$, Pearson's $r$), indicating how they were calculated |

*Our web collection on statistics for biologists contains articles on many of the points above.*

## Software and code

Policy information about availability of computer code

| | |
|---|---|
| Data collection | LAS X software 3.2 https://www.leica-microsystems.com/products/microscope-software/<br>Zen software 1.1 https://www.zeiss.com/microscopy/int/products/microscope-software/zen.html |
| Data analysis | Zen software 1.1 https://www.zeiss.com/microscopy/int/products/microscope-software/zen.html<br>Graphpad Prism 9.0 Graphpad software https://www.graphpad.com<br>ImageJ software 1.53 https://imagej.net/software/imagej1 |

For manuscripts utilizing custom algorithms or software that are central to the research but not yet described in published literature, software must be made available to editors and reviewers. We strongly encourage code deposition in a community repository (e.g. GitHub). See the Nature Portfolio guidelines for submitting code & software for further information.

## Data

Policy information about availability of data

All manuscripts must include a data availability statement. This statement should provide the following information, where applicable:

- Accession codes, unique identifiers, or web links for publicly available datasets
- A description of any restrictions on data availability
- For clinical datasets or third party data, please ensure that the statement adheres to our policy

Authors can confirm that all relevant data are included in the paper and/or its supplementary information files. Source data are provided with this paper.

# Field-specific reporting

Please select the one below that is the best fit for your research. If you are not sure, read the appropriate sections before making your selection.

☒ Life sciences ☐ Behavioural & social sciences ☐ Ecological, evolutionary & environmental sciences

For a reference copy of the document with all sections, see nature.com/documents/nr-reporting-summary-flat.pdf

# Life sciences study design

All studies must disclose on these points even when the disclosure is negative.

| | |
|---|---|
| Sample size | Sample sizes were chosen based on similar published studies, with at least n=3 for biological samples of LLPS. Ref:Tang, L. Liquid phase separation. Nat Methods 16, 18 (2019). |
| Data exclusions | No data were excluded. |
| Replication | All the experiments were repeated at least three times with consistent data. |
| Randomization | Randomization is not relevant for this study because our work does not involve clinical trials or population studies. The biological samples of proteins or cells were not distinguishable for experiment performers. |
| Blinding | Blinding is not relevant for this study because no group allocation was performed. |

# Reporting for specific materials, systems and methods

We require information from authors about some types of materials, experimental systems and methods used in many studies. Here, indicate whether each material, system or method listed is relevant to your study. If you are not sure if a list item applies to your research, read the appropriate section before selecting a response.

## Materials & experimental systems

| n/a | Involved in the study | |
|---|---|---|
| ☐ | ☒ | Antibodies |
| ☐ | ☒ | Eukaryotic cell lines |
| ☒ | ☐ | Palaeontology and archaeology |
| ☒ | ☐ | Animals and other organisms |
| ☒ | ☐ | Human research participants |
| ☒ | ☐ | Clinical data |
| ☒ | ☐ | Dual use research of concern |

## Methods

| n/a | Involved in the study | |
|---|---|---|
| ☒ | ☐ | ChIP-seq |
| ☒ | ☐ | Flow cytometry |
| ☒ | ☐ | MRI-based neuroimaging |

## Antibodies

Antibodies used

FGFR1 Antibody (clone: M17D10) [Alexa Fluor® 405] (Novus, NB100-2080AF405, 1:1000);
Heparan Sulfate antibody (10E4 epitope, clone: 8.S.087) [biotin] (USBiological, H1890-01, 1:100)
Goat Anti-Mouse IgM (μ chain specific, polyclonal) [Alexa Fluor 647] (Jackson, 115-605-020, 1:200)
p-ERK 1/2 Antibody (pT202/pY204.22A) (Santa Cruz, sc-136521, 1:100)
ERK 1/2 Antibody (MK1) (Santa Cruz, sc-135900, 1:200)
Vinculin Antibody (7F9) (Santa Cruz, sc-73614, 1:200)
Goat Anti-Mouse IgG (H+L) [HRP] (Beyotime, A0216, 1:2000)
Anti-Rab4 antibody[EPR3043] (abcam, ab109009, 1:170)
Cy3-labeled Goat Anti-Mouse IgG(H+L) (Beyotime, A0521, 1:500)

Validation

FGFR1 Antibody (Novus, NB100-2080AF405) https://www.novusbio.com/products/fgfr1-antibody-m17d10_nb100-2080af405;
Heparan Sulfate antibody (USBiological, H1890-01) https://www.usbio.net/antibodies/H1890-01/heparan-sulfate-10e4-epitope-biotin;
Goat Anti-Mouse IgM (Jackson, 115-605-020) https://www.jacksonimmuno.com/catalog/products/115-605-020;
p-ERK 1/2 Antibody (Santa Cruz, sc-136521) https://www.scbt.com/p/p-erk-1-2-antibody-pt202-py204-22a;
ERK 1/2 Antibody (Santa Cruz, sc-135900) https://www.scbt.com/p/erk-1-2-antibody-mk1;
Vinculin Antibody (Santa Cruz, sc-73614) https://www.scbt.com/p/vinculin-antibody-7f9;
Goat Anti-Mouse IgG (Beyotime, A0216) https://www.beyotime.com/product/A0216.htm
Anti-Rab4 antibody[EPR3043] （abcam, ab109009) https://www.abcam.cn/rab4-antibody-epr3043-early-endosome-markerab109009.
html
Cy3-labeled Goat Anti-Mouse IgG(H+L) (Beyotime, A0521) https://www.beyotime.com/product/A0521.htm

## Eukaryotic cell lines

Policy information about cell lines

| | |
|---|---|
| Cell line source(s) | Mouse embryonic fibroblast (MEF) cells were purchased from National infrastructure of cell line.<br>Neuro-2a (N2a) cells were obtained from Tsinghua University, originally purchased from National infrastructure of cell line.<br>CHO-K1 and CHO-677 cells were purchased from GlycoNovo Technologies, Shanghai. |
| Authentication | Cell line authentication was not performed. |
| Mycoplasma contamination | The cell lines were not tested for mycoplasma contamination. |
| Commonly misidentified lines<br>(See ICLAC register) | No commonly misidentified cell lines were used in this study. |
