# Supplementary figures and images for: Phase separation on cell surface facilitates bFGF signal transduction with heparan sulphate
Source: Nat Commun. 2022 Mar 2;13:1112. doi: 10.1038/s41467-022-28765-z (PMC8891335; doi:10.1038/s41467-022-28765-z)

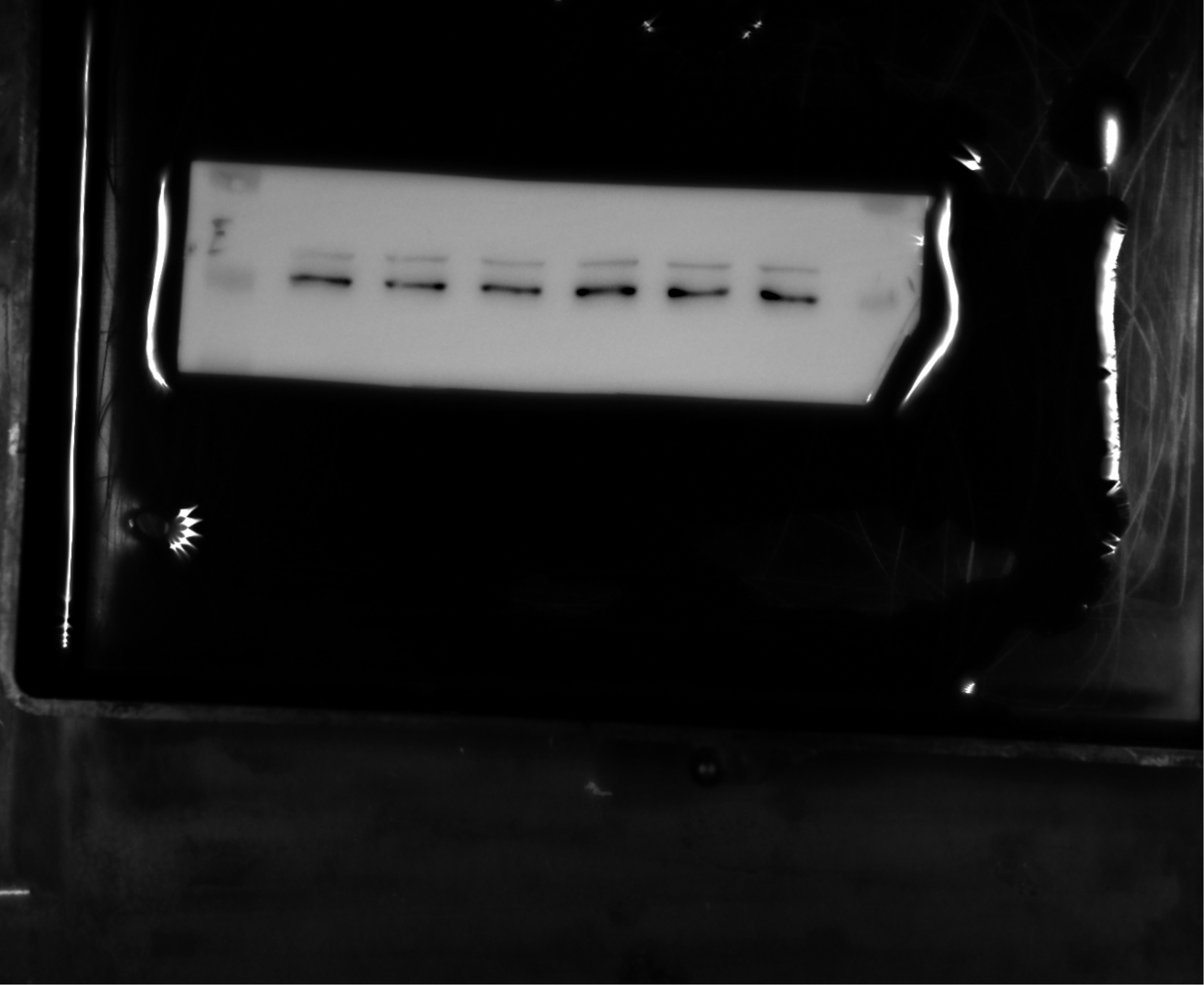

Supplement: Supplementary file 3 — Source Data [file 41467_2022_28765_MOESM3_ESM.zip › Western Blot Raw/erk.jpg]

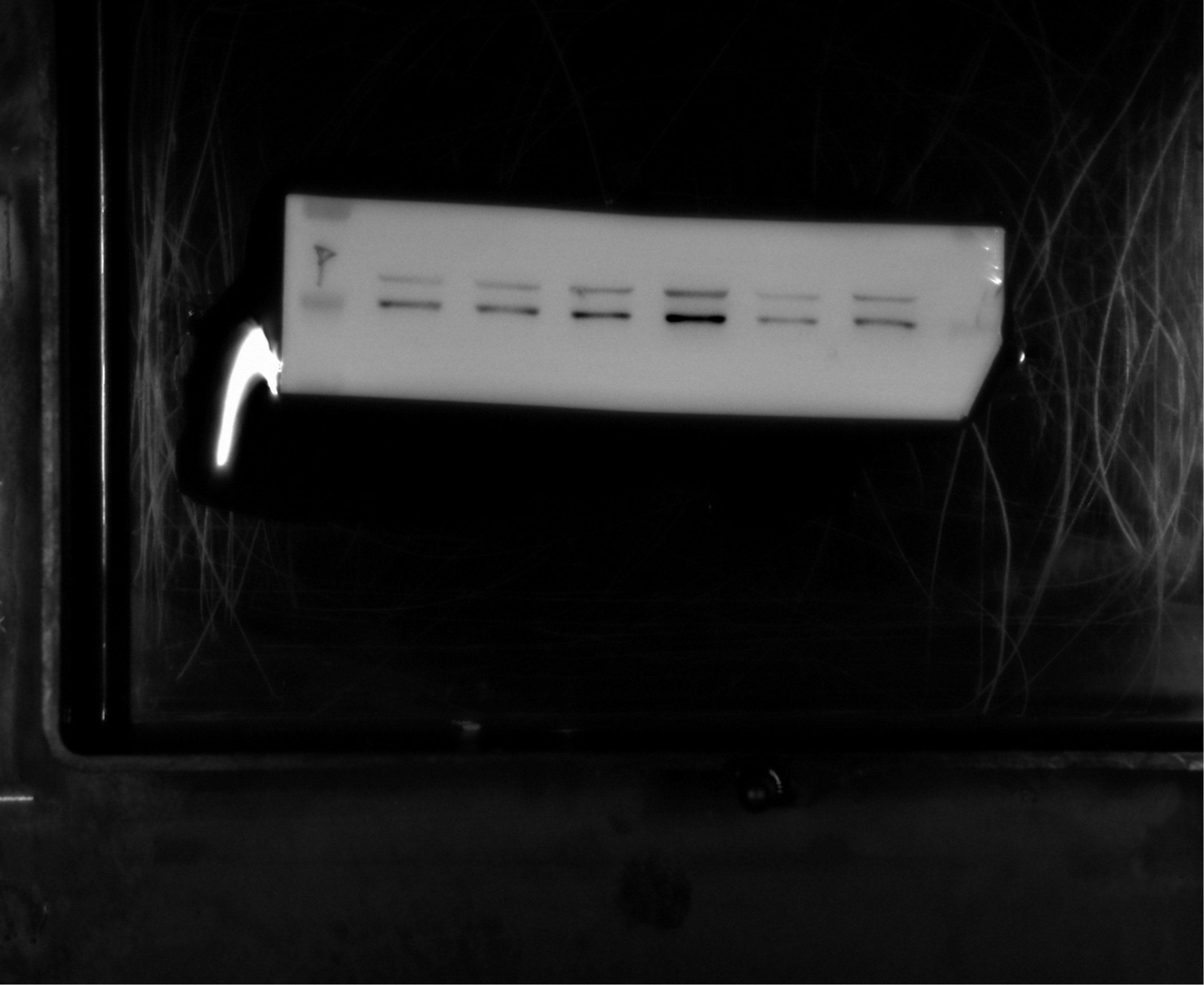

Supplement: Supplementary file 3 — Source Data [file 41467_2022_28765_MOESM3_ESM.zip › Western Blot Raw/p-erk.jpg]

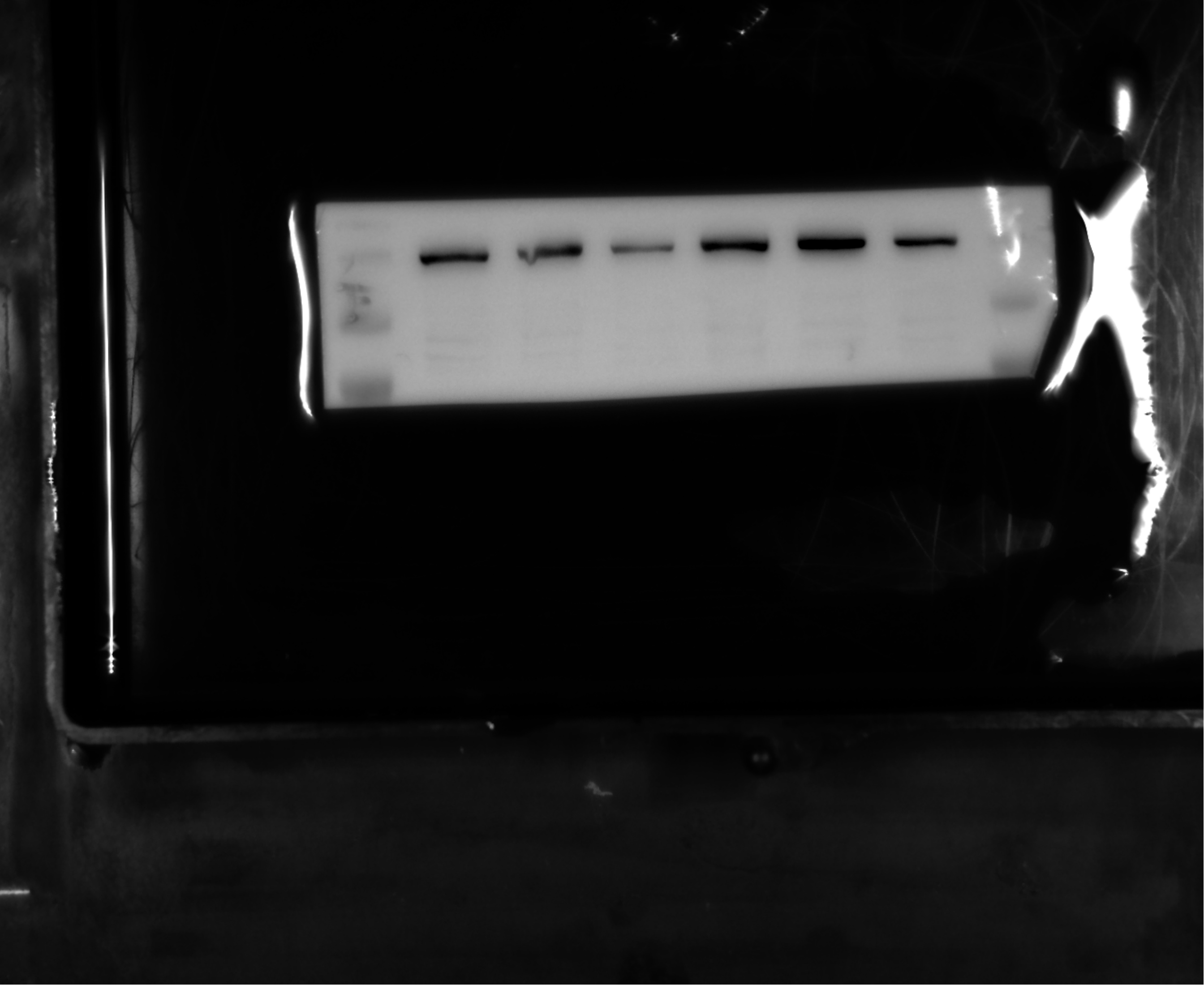

Supplement: Supplementary file 3 — Source Data [file 41467_2022_28765_MOESM3_ESM.zip › Western Blot Raw/vinculin-erk.jpg]

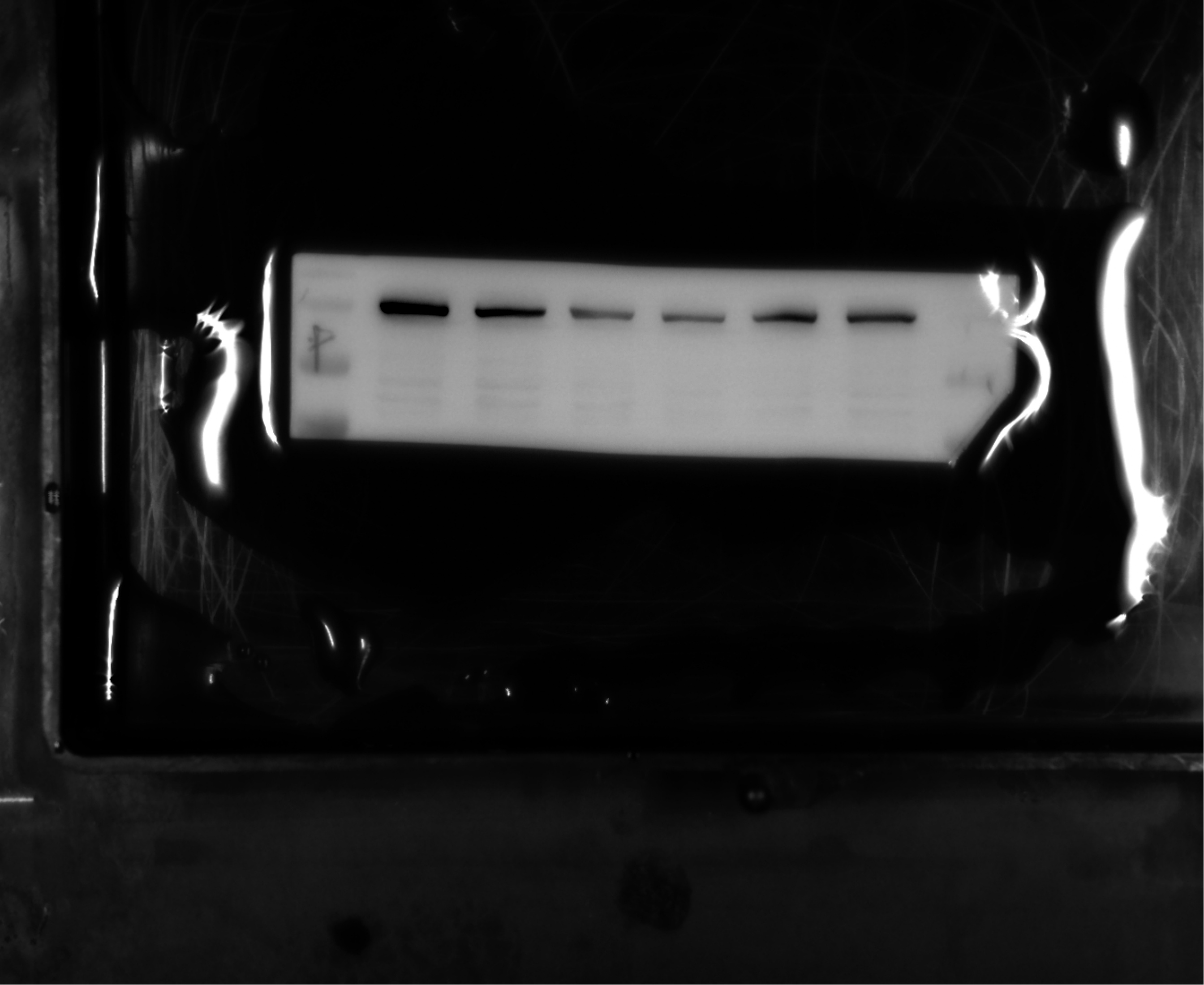

Supplement: Supplementary file 3 — Source Data [file 41467_2022_28765_MOESM3_ESM.zip › Western Blot Raw/vinculin-p-erk.jpg]
